# Developing and piloting a multifactorial intervention to address participation and quality of life in nursing home residents with joint contractures (JointConImprove): study protocol

**DOI:** 10.3205/000217

**Published:** 2015-07-15

**Authors:** Martin Müller, Gabriele Bartoszek, Katrin Beutner, Hanna Klingshirn, Susanne Saal, Anna-Janina Stephan, Ralf Strobl, Eva Grill, Gabriele Meyer

**Affiliations:** 1 Institute of Medical Information Processing, Biometrics and Epidemiology, Ludwig-Maximilians-Universität München, Munich, Germany; 2 German Center for Vertigo and Balance Disorders, Ludwig-Maximilians-Universität München, Munich, Germany; 3 Faculty of Health, School of Nursing Science, Witten/Herdecke University, Witten, Germany; 4 Institute of Health and Nursing Science, Martin Luther University of Halle-Wittenberg, Halle, Germany; 5 Munich Center of Health Sciences – MCHealth, Ludwig-Maximilians-Universität München, Munich, Germany

**Keywords:** contracture, aged, aged 65 and over, disabled persons, complex intervention, participation, quality of life, geriatric rehabilitation, nursing homes, long-term care

## Abstract

**Background:** Joint contractures are common problems in frail older people in nursing homes. Irrespective of the exact extent of older individuals in geriatric care settings living with joint contractures, they appear to be a relevant problem. Also, the new emphasis on the syndrome of joint contractures, e. g. by the German statutory long term care insurance, led to an increase in assessment and documentation efforts and preventive interventions in clinical care. However, more attention should be paid to the actual situation of older individuals in nursing homes with prevalent joint contractures, particularly their experience of related activity limitations and participation restrictions. Thus, the aim of this study is 1) to develop a tailored intervention to improve functioning, and especially participation and quality of life in older residents with joint contractures in nursing homes and 2) to test the feasibility of the intervention accompanied by a rigorous process evaluation.

**Methods:** The complex intervention, which will be developed in this project follows the UK Medical Research Council (MRC) framework and integrates the perspectives of all potentially relevant user groups, from the affected individuals to clinicians and researchers. The development process will comprise a systematic literature review, reanalysis of existing data and the integration of the knowledge of the affected individuals and experts. The developed intervention including a comprehensive process evaluation will be pilot tested with residents with joint contractures in three nursing homes.

**Discussion:** The projected study will provide a tailored intervention to improve functioning, participation and quality of life in older residents with joint contractures in nursing homes. With this focus, the intervention will support patient relevant outcomes. The pilot study including process evaluation will offer a first opportunity to indicate the size of the intervention’s effect and prepare further studies.

## Zusammenfassung

**Hintergrund:** Gelenkkontrakturen sind häufige Probleme gebrechlicher älterer Menschen in Pflegeheimen. Unabhängig von der genauen Anzahl an älteren Menschen, die an Gelenkkontrakturen leiden, scheint dieses Syndrom ein relevantes Problem im Setting Pflegeheim dazustellen. Durch einen zunehmenden Fokus auf Gelenkkontrakturen, z.B. durch die Pflegeversicherung, kam es zu einem Anstieg im Dokumentations- und Assessmentaufwand und in der Einführung von Präventionsmaßnahmen. Viel mehr Aufmerksamkeit sollte aber auf die tatsächliche Situation der älteren Menschen mit Gelenkkontrakturen in Pflegeheimen gelegt werden, vor allem deren tatsächlichen Einschränkungen in Aktivitäten und Teilhabe. Das Ziel dieser Studie ist daher, 1) die Entwicklung einer maßgeschneiderten Intervention zur Verbesserung der Funktionsfähigkeit, sozialen Teilhabe und Lebensqualität von Menschen mit Gelenkkontrakturen in Pflegeheimen und 2) die Überprüfung der Machbarkeit der Intervention, begleitet von einer Prozessevaluation.

**Methoden: **Die Entwicklung der komplexen Intervention folgt dem UK Medical Research Council (MRC) Framework und integriert die Perspektiven aller potenziell relevanten Benutzergruppen von den betroffenen Personen über Kliniker und Forscher.

Der Entwicklungsprozess beinhaltet einen systematischen Literaturreview, die Re-Analyse vorhandener Daten, Fokusgruppeninterviews mit Betroffenen, eine Expertentagung und eine Delphi-Studie mit klinischen Experten sowie eine Cluster-randomisierte Pilotstudie mit umfassender Prozessevaluation.

**Diskussion:** Die geplante Studie wird eine maßgeschneiderte Intervention zur Verbesserung von Funktionsfähigkeit, sozialer Teilhabe und Lebensqualität von Menschen mit Gelenkkontrakturen in Pflegeheimen bereitstellen. Die Pilotstudie inklusive der Prozessevaluation stellt einen ersten Schritt zur Schätzung der Stärke des Interventionseffektes dar und wird weitere Studien vorbereiten.

## Background

Joint contractures are associated with restrictions in full range of motion of any joint due to deformity, disuse, and pain. They are common problems of frail older people, particularly in nursing home residents [1], [2]. Irrespective of the various causes and underlying health conditions, e.g. neurological or musculoskeletal conditions, joint contractures can have significant impact on overall quality of life and functioning of the affected individuals, limit daily activities and mobility and lead to other negative consequences, such as pain or increased risk of falls [3], [4], [5], [6], [7]. 

Prevalence data on joint contractures are scarce and estimates vary widely, presumably due to different definitions and populations. Studies involving nursing home residents in the US report prevalence rates between 25% [8] and 80% [2], [3], [6], [9], [10]. 

Irrespective of the exact number of older individuals in geriatric care settings living with joint contractures, joint contractures appear to be a relevant problem and receive growing attention. For example, the German federal statutory long term care insurance system has defined the assessment of risk for joint contractures and the implementation of preventive measures as quality indicators in long-term nursing care [11], [12], [13]. This new emphasis on the syndrome of joint contractures led to an increase in assessment and documentation efforts and preventive interventions in clinical care [14], even though evidence for the effectiveness of preventive measures is lacking [15], [16], [17]. 

As proofs of effectiveness are missing for the primary and secondary preventive interventions that are part of the current standard of care in joint contractures, an alternative focus should be on interventions which do not merely address prevention or treatment of joint contractures but also focus on maintaining or enhancing participation and quality of life. Thus, more attention should be paid to the actual situation of older individuals in nursing homes with prevalent joint contractures, particularly their experience of related activity limitations and participation restrictions. To address this issue, we have recently developed a standard set based on the WHO’s International Classification of Functioning, Disability and Health (ICF) [18] to comprehensively describe activity limitations and participation restrictions and are developing a standardized assessment instrument to quantify the extent of activity limitations and participation restrictions [19]. Based on the results of the preceding project the next step is now to develop an intervention to address the actual situation of older individuals in nursing homes with prevalent joint contractures. Hence, the aim of this study is 1) to develop a tailored complex intervention to improve functioning, and especially participation and quality of life in older residents with joint contractures in nursing homes and 2) to test the feasibility of the intervention and the process evaluation tools in a pilot study. 

## Methods

The development of relevant, effective and feasible health care interventions is challenging, since changes in health care practice are rather complex than deterministic. To address these issues und to ensure the development of theoretically and empirically plausible, consumer-orientated and feasible interventions, the UK MRC framework was introduced to provide guidance on how to develop and evaluate complex interventions. It identifies the following four key elements of this potentially cyclical process: development, feasibility/piloting, implementation and evaluation. This study protocol focuses on the development and the feasibility/piloting phases. The development phase usually includes identification of the relevant evidence base, identification or development of a theory and modelling of the intervention process and the addressed outcomes. In the feasibility and piloting stage, the acceptability and feasibility of the developed intervention is carefully assessed. Also, recruitment procedures and recruitment and retention rates have to be evaluated. In addition, the basis for sample size calculations can be investigated in this phase [20], [21]. Based on this framework, our study has a design with seven successive steps. 

An overview of all applied methods and designs is given in Table 1 (Tab. 1). 

The study protocols of all empirical parts of the projects will be presented to the respective ethics committees of the Ludwig-Maximilians-Universität in Munich and the Martin-Luther-University in Halle-Wittenberg prior to the start of the respective part. Written informed consent from the participants or their legal guardians will be obtained prior to inclusion in the study.

### (1) Identification of evidence (development phase): Systematic Review

**Aims: **The aim of this systematic review is to identify the existing evidence regarding prevention and/or treatment of disability due to joint contractures which serves as basis for the intervention modelling. 

**Methods: **The systematic review will be carried out in electronic databases (PUBMED, CINAHL, EMBASE, Psychinfo) and via hand searches. The process and results will be documented using an electronic database. The review will include randomized and non-randomized controlled trials on programs for prevention and/or treatment of disability due to acquired joint contractures. Target population are individuals in need for long term care of either type. 

**Analysis:** The critical appraisal will follow the Cochrane Handbook for Systematic Reviews of Interventions, version 5.1.0 [22]. Since retrieved studies are expected to be too diverse to be analysed by means of a meta-analysis, a narrative synthesis as suggested by the York University Centre for Reviews and Dissemination ([23], p. 48) will be carried out. A detailed description of the review protocol can be found at PROSPERO (http://tinyurl.com/JCISystRev).

### (2) Identification of intervention goals (development phase): Graphical model

**Aims: **The aim of the graphical models is to identify ICF categories which could serve as intervention targets. Graphical models depict association structures between variables with complex interactions and differentiate confounding variables from intermediate variables [24], [25], [26]. Graphical models lead to network-like graphs in which hubs, i.e. nodes with several connections to other nodes, can be identified. By addressing such hubs by targeted interventions, many other categories may be influenced. 

**Participants and design: **We will apply graphical modelling to existing data on prevalence of functioning and disability in patients with joint contractures [7], [27]. In brief, the study sample to be analysed consists of a cross-sectional sample of 299 older patients (>65 years) with joint contractures in geriatric care settings (acute and post-acute specialized rehabilitation, nursing home). Functioning and disability was appraised by 124 categories of the International Classification of Functioning, Disability and Health. Among those, 28 categories referred to the ICF component body functions, 80 to activities and participation and 16 to environmental factors. Data will be dichotomized in “no problem” vs. “any problem”.

**Analysis:** Since graphical models are susceptible to small changes in the data set leading to great changes in the result, we implement bootstrap aggregating (bagging) to stabilize the outcome of such a model and subsequently enhance accuracy [28], [29], [30] and validity of the results. Multiple imputations will be used to adequately handle missing values in our data set [31]. To develop a graphical model, the conditional independencies will be identified via the neighbourhood of a variable. The neighbourhood of a variable is defined as the set of conditionally dependent variables. Meinshausen and Buehlmann [32] showed that neighbourhood selection by means of the so-called least absolute shrinkage and selection operator (LASSO) [33] is superior to common methods, in particular, if the number of variables exceeds the number of observations. The set of predictor variables corresponding to nonzero coefficients in a prediction model represents the neighbourhood. By estimating the neighbourhood for each variable the whole graphical model can be identified. 

### (3) Identification of intervention goals (development phase): Focus groups

**Aims:** To also incorporate the consumer perspective, focus group interviews with nursing home residents affected by joint contractures will be carried out as a complimentary approach to the data driven identification of potential intervention goals by graphical models. In the focus groups, desirable goals and potential facilitators and barriers to reach these goals will be discussed [34]. 

**Participants and design:** The focus groups with residents will take place in the cooperating nursing homes. To ensure adequate communication, a maximum of five participants are feasible since they will be of old age and presumably frail. Therefore, four focus groups resulting in a sample size of approximately 20 participants will be planed. Final sample size will be determined by saturation. Saturation refers to the point at which an investigator has obtained sufficient information from the field [35]. The focus groups with affected individuals [34] will follow quantitative descriptive methodology [36]. A possible source of bias for the focus groups is the recruitment of participants, which have to be able to participate and to communicate adequately. This selection could introduce bias towards individuals with milder disease and less severe problems. Even with the risk of bias in mind, focus groups are able to result in more comprehensive data than individual interviews [37]. This is especially true for those individuals planned to be included in our study who share the same living environment and associated barriers and facilitators because they might put each other in mind of relevant aspects. In addition, experiences from prior individual interview studies with nursing home residents with joint contractures indicate feasibility [38]. 

**Analysis:** The results of the focus groups will be categorized using content analysis according to the concept of qualitative description [36], [39] by two independent researchers. 

### (4) Identification and development of intervention components (development phase): Expert panel

**Aims:** To develop a preliminary list of intervention components, a structured two-day meeting with renowned national clinical experts will be held. 

**Participants and design:** The results of the systematic review, the potential intervention goals and the focus groups as well as the results of the qualitative interviews from our previous project [38] will be presented. The results of the qualitative interviews will be introduced at this point to further address the patients’ perspective. The expert consultation will be organized as a formal consensus building process where a group of experts will engage in plenary discussion and smaller working groups. All steps of the consensus process will be documented in an electronic database. The expert panel will be constituted by inviting outstanding national experts in the field of geriatric care and medicine and will comprise about 10 persons. 

### (5) Identification and development of intervention components (development phase): Expert Delphi survey

**Aims:** The results from step 4 will be further validated in an expert Delphi survey. 

**Participants and design:** The potential intervention components will be evaluated and missing aspects can be mentioned in up to three rounds [40]. The survey will be carried out via an online platform and will involve health professionals from all relevant professions (nurses, physicians, physical therapists, occupational therapist) and all relevant fields (nursing homes, geriatric rehabilitation, acute care hospitals). Inclusion criteria will be clinical expertise (work experience >5 years in geriatric care settings, i.e. hospitals, rehabilitation facilities and nursing homes). The experts will be selected from an existing data base from the previous project where they have committed for participation. Further sources of contacts are both suggestions from other experts and professional organizations and identification through authorship of relevant publications. Literature on Delphi surveys suggests a group size of about 20 [41]. We will carry out a separate survey for physicians, nurses and therapists. 

**Analysis: **The results of the Delphi survey will be categorized using content analysis according to the concept of qualitative description [36], [39] by two independent researchers. 

### (6) Piloting of the complex intervention (feasibility and piloting phase): Pilot study

**Aim:** The main objective of the pilot study is to estimate the effect size in order to plan the sample size for the main cluster-randomized trial. 

**Participants and design:** The pilot study will be designed as a cluster randomized controlled study to assess the feasibility of the developed intervention and its components. Target population for the pilot study will be residents with joint contractures in six nursing homes in the area of Munich, Halle and Witten. Presence of joint contractures will be evaluated by either physicians’ diagnosis or the diagnosis of a skilled staff nurse or physical therapist. There will be no other inclusion criteria. We will exclude residents with limited life expectancy due to advanced cancer or other diseases with poor prognosis according to the assessment by the nurse in charge. Each nursing home will be randomly assigned to the intervention group or to the control group. In line with suggestions of Billingham et al. [42] we will include a total sample size of about 120 residents, 60 in the intervention and 60 in the control group.

The primary outcome will be the impact of joint contractures on functioning and participation as measured by a recently developed instrument [27]. This outcome measure consists of a selection of categories of the International Classification of Functioning, Disability and Health (ICF), operationalized in a consensus meeting among clinical experts. This selection is currently being validated according to its psychometric properties with an emphasis on specific objectivity in the sense of Rasch analysis [43]. This Rasch-validated instrument will be used as primary outcome measure. Secondary outcomes will be collected using established instruments fitting the intervention components.

We also collect gender, age, socioeconomic variables, activities of daily living through Barthel Index [44], Mini Mental State Examination [45], and presence of chronic diseases [46] as potential confounders.

**Analysis:** The effects of the intervention over 6 months will be analysed on an intention-to-treat basis. Group differences will be assessed by mean and standard deviation. In addition, the influence of different nursing home characteristics (e.g. staffing characteristics, ward size etc.) will be assessed using multiple regression models. Also results of the process evaluation will be taken into account when interpreting the results of the pilot study. 

### (7) Piloting of the complex intervention (feasibility and piloting phase): Process evaluation 

**Aims:** To evaluate the context of the trial and to learn from the factors which are associated with successful or failing implementation of the intervention components, a process evaluation will be carried out alongside the pilot study. Also, it is intended to use the process evaluation of the pilot study to prepare the process evaluation of the subsequent main trial. 

**Participants and design:** This process evaluation will follow the framework provided by Grant et al. [47] and will address recruitment, reach and fidelity of the intervention, satisfaction of users, staff and organizations. Also, the data collection procedures of this pilot study will be used to test instruments and methods required for recording the cost data relevant for an economic evaluation alongside the main trial. The process evaluation will use quantitative and qualitative methods (numeric evaluation of recruitment and reach via checklists and scales, individual interviews with residents and focus groups for the nursing and therapeutic staff and the management of the nursing homes). The aim to minimize bias in the process evaluation will be reached by implementing different aspects of data collection with the use of standardized instruments, focus group interviews, quantitative and qualitative methods. 

**Analysis:** Results of the process evaluation will be analysed with descriptive statistics and qualitative descriptive methodology [36], [39].

To improve overall scientific quality, an external advisory board with clinicians and methodological experts will be established.

## Discussion

This study protocol describes the development and piloting of a complex intervention that aims to improve the situation of older individuals with joint contractures in nursing homes. With the emphasis on participation and quality of life, this intervention will address a health outcome that is highly relevant to the affected individuals. This is particularly important since interventions aiming to improve the physical perspective of joint contractures (i.e. range of motion) seem to have limited effects [15], [16], [17]. 

Developing complex interventions is a challenging task [48]. Unfortunately, many randomized controlled trials dealing with complex interventions did not consider a stepwise developmental process covering theory funding, modelling of intervention components and outcome parameters and piloting before experimental implementation [21], [49]. Sometimes, failure to improve clinical outcomes or non-expected outcomes cannot be explained and investigators raise questions at the end of their trial which clearly should have been answered before the experimental study (e. g. [50]). Often there was no process evaluation; thus barriers and facilitators of the intervention remain unexplained [51]. From an ethical and economical point of view, failure to succeed with a complex intervention should be reduced as far as possible by spending more effort on its systematic and careful development and evaluation. Our project is designed to resolve these issues and will end up with a thoroughly evaluated intervention that will have to prove its effectiveness in a large scale cluster randomized controlled trial in the future. Eventually, there should be a long-term implementation of the intervention in different geriatric care settings to increase the standard in nursing care. 

## Notes

### Competing interests

The authors declare that they have no competing interests.

### Authors’ contributions

MM, EG and GM contributed to the conception of the study and applied for funding. MM, EG and GM conceived the study design. MM and HK drafted the manuscript. SS, AS, KB, GM, EG, GB, and RS critically revised the drafts and contributed to the final writing of the paper. EG and GM share the senior authorship. All authors read and approved the final manuscript. 

### Funding

The project is funded by the German Federal Ministry of Education and Research (01GY1327A and 01GY1327B – JointConImprove).

## Figures and Tables

**Table 1:**
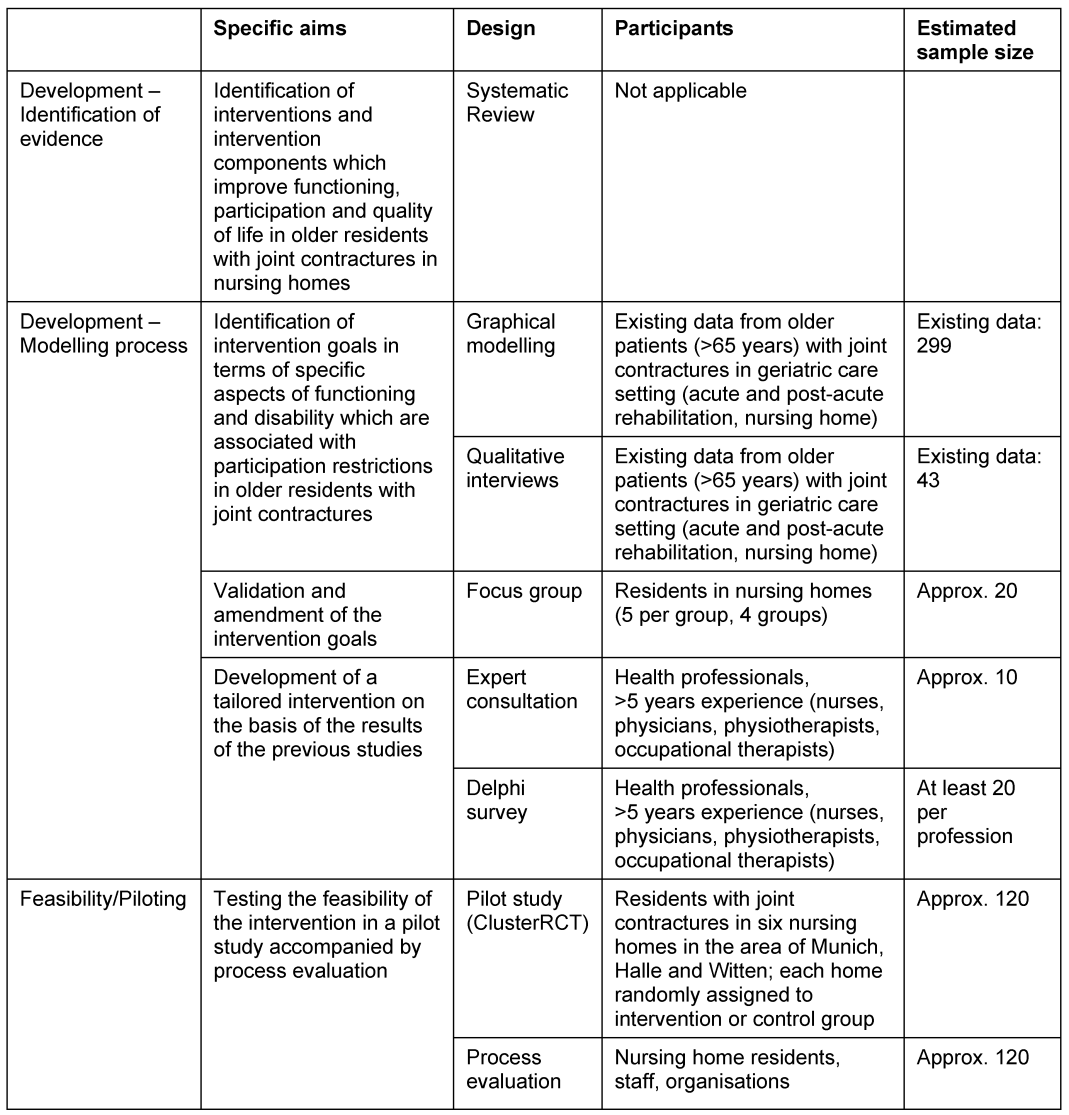
Overview of the study parts
